# Cellular Mechanisms of Electromagnetic Field in Traumatic Brain Injury

**DOI:** 10.26502/jbb.2642-91280075

**Published:** 2023-04-07

**Authors:** Imran Siddiqi, Maxwell Marino, Devendra K. Agrawal, Dave Baron, David A. Connett, Daniel Miulli

**Affiliations:** 1 Riverside University Health System, Department of Neurological Surgery, USA; 2 Department of Translational Research, College of Osteopathic Medicine of the Pacific, Western University of the Health Sciences, Pomona, California; 3 Department of Surgery, Arrowhead Regional Medical Center, Colton, California

**Keywords:** Anti-inflammation, Brain ischemia, Brain trauma, Concussion, Electromagnetic field, Excitotoxicity, Inflammasomes, Multiple Brain Impacts, Neuroinflammation, Nitric Oxide, Oxidative stress, Reactive oxygen species, Stroke

## Abstract

This paper presents a comprehensive review of the extant literature from 1980 through 2023 on the role and utility of Electromagnetic Fields (EMF) in the treatment of brain trauma and brain neuropathology resulting from disease. Brain trauma resulting from accident, injury and disease is a significant contributor to short and long-term morbidity, as well as a leading cause of mortality globally. To date, limited effective treatments strategies exist, and are focused primarily on symptom relief, not restoring primary preinjury function and structure. Much of the current clinical literature is based on retrospective case reports and limited animal model prospective trials exploring core etiology and alterations in post-injury clinical phenotypes. The current findings reported in the scientific literature suggest that electromagnetic therapy may hold promise as a potential non-invasive treatment for traumatic brain injury and neuropathology. Although promising, well designed clinical trials are needed to better determine its potential clinical effectiveness in this diverse patient population. Future trials will need to determine the impact of clinical variables, such as sex, age, type and extent of injury and pathology, pre-injury baseline health status and a comprehensive biopsychosocial assessment to determine a more effective personalized approach to patient care. Although initially showing promise, much work needs to be done.

## Introduction

The utility of Electromagnetic Fields (EMF) in the measurement and treatment of various disease states is a novel and progressive area of medical research. An emerging body of basic and clinical knowledge is growing and evolving. As patients are afflicted by serious neuropsychiatric pathology, such as brain trauma, stroke, and CNS tumors, the limitations of conventional treatments, such as surgery or pharmaceuticals is under ongoing clinical scrutiny for short and long-term effectiveness. The advent of new less invasive treatment options is necessary and offers hope for an improved quality of life for current and future patients. Our research group has been exploring a novel treatment of brain pathologies, such as traumatic injury, stroke, and tumor, resulting from technological advancements in Electromagnetic Field (EMF) research and its application in the clinical setting. An enhanced understanding of how cellular and molecular mechanisms, particularly in neurons and brain tissue, are affected by Traumatic Brain Injury (TBI) and how modulation of their cellular processes may be achieved with EMF technology is important. The physical properties of electrons, protons, and molecular structures in biology are well studied and translating this knowledge to clinical situations utilizing technologies such as ultrasound and Magnetic Resonance Imaging (MRI) has offered valuable insights into etiology of core clinical symptoms. In addition, EMF in the form of Pulsed Electromagnetic Field (pEMF) is used in spinal cord stimulators to relieve pain, in bone stimulators to heal non unions, in tremors to calm the hands, and in the heart to capture a beat. The refinement of these clinical applications comes with multidisciplinary integration of expertise and knowledge in pathology, physiology, chemistry, biology, physics, clinical medicine, and biomedical engineering. This review aims to critically review the underlying cellular and molecular mechanisms that result as a response to brain injury and those that are responsible for the healing and modulation of physiological parameters of nervous tissue. This understanding is critical in determining the role of EMF as a clinical tool for promoting healing and hopefully one day serving as a modality of improving outcomes in patient care experiencing brain pathology.

## Methods

A comprehensive literature search utilizing PubMed and Google Scholar was conducted between January 1980 through February 2023. Articles published in English were reviewed and included in this article. The following key words were utilized: EMF stroke, electromagnetic field, cerebrovascular accident, pulsed electromagnetic field (PEMF), EMF and traumatic brain injury, brain trauma, neuroinflammation, neuroprotection and EMF, immunomodulation, and various iterations. The findings were critically reviewed, and the information was synthesized into this comprehensive review article highlighting the clinical application and future directions of EMF on cellular pathophysiology.

## Discussion

### Electromagnetic Properties of Neurons

At the cellular level, the action potential occurs over 1 millisecond (ms) (1000 Hertz (Hz)); it is hyperpolarized for 2–3 ms (300 Hz) at +15 millivolt (mV) with a relative refractory period of 15 ms, yielding a relative action potential generation rate of 66.6 Hz. Post-synaptic potentials arrive at a frequency of 1000 Hz. Cell maintenance occurs at 30–5000 Hz. Using clinical micro-recording devices, the frequencies of groups of cells have been discovered. Nordhausen developed a 3D silicon electrode array which records 100 separate channels of neuronal activity [1]. Rousche and Normann reported that this same type of array called the Utah Intracortical Electrode Array stimulates and records in a single layer up to 1.5 millimeter (mm) beneath the surface [2]. Neuronal cells exchange information at different rates: cochlear cells have a frequency of 1000–4000 Hz at −20 to −80 mV, subthalamic nuclei 37 Hz, globus pallidal external cells 40 Hz, substantial nucleus reticulata 75 Hz, and globus pallidal interna cells 80–90 Hz. Neuronal activity is a macrocosm of types of activity at the atomic level. Individual atoms are known to vibrate at individual Larmor frequencies. The frequencies at 1 Tesla (T) are H+ 42.6 Megahertz (MHz), H2 6.5 MHz, P31 17.2 MHz, C13 10.7 MHz, N14 3.1 MHz, N15 4.3 MHz, S33 3.3 MHz, F19 40.1 MHz. These frequencies are detected and “tuned in” by an MRI. One of the most sensitive detectors is our retina, it detects photons at a frequency of 1015 Hz.

### Groups of Cells

Frequencies from groups of cells have also been studied. Modulation of signals occurring in the Ventral Intermediate Nucleus (VIM) from 130–150 Hz arrests motor activity and at 10–500 Hz arrests sensory activity. According to Benabid, stimulation greater than 100 Hz inhibits VIM, subthalamic nucleus (STN) and globus pallidus internus (GPi) [3]. Specifically, 130 Hz, 50 msec pulse width, suppresses activity of the STN and inhibits glutamate, possibly being neuroprotective. However, under pathological conditions, the dysfunctional penumbra of the cortex fires at 700 Hz for several days. This is due to the spreading depression involving the glial potassium current. In the penumbra of the thalamus the dysfunctional firing occurs at 900 Hz [4]. The Gpi can be stimulated to contract the face at 330 Hz, 300 msec pulse width and 50 mA. VEP external stimulus, thus to the retina, occurs at 1.1 Hz and 200–400 msec. Dorsal column, spinal cord stimulation, takes place across the dura at 4.3 V, 50–85 Hz and 200–450 msec pulse width. There are anecdotal reports of severely head-injured patients awaking in MRI scanners during image acquisition. Interestingly, the majority occurs in 1.5 Tesla (T) units that have a RF frequency of 4kHz. The penetration into the brain tissue of the radiofrequency (RF) signal, is a function of the frequency, such that keeping constant power a 1 MHz RF signal penetrates greater than 11 cm, 41 MHz 11 cm, and 100 MHz 7 cm [5]. Two methods for activating cells in the motor cortex utilizing PEMF have been developed. This technique does not require direct contact with the surface of the brain. In 1980, Merton and Morton introduced the first method, transcranial electrical stimulation (TES) [6]. It utilizes a high-voltage electrical pulse (up to 2 kV) of a short duration (less than 10 us) delivered through electrodes placed on the scalp with the anode over the cortical region to be stimulated (anode stimulation). In 1985, Barker and Co-workers first reported the other form of noninvasive cortical cell activation, Transcranial Magnetic Stimulation (TMS) [7]. They used a high-current (4 kA) and short-duration (110 msec) pulse width. This was discharged through a coil placed on the scalp over the motor strip to produce a brief magnetic field, resulting in secondary currents inside the brain, which then depolarized cells within the motor cortex. For a given magnetic pulse, the direction of current flow through the coil, as well as the shape and size of the coil, play important roles in determining the effectiveness of TMS [8]. The TMS and TES are the best clinical approaches to stimulate the organelle, cell, and tissue. This occurs in the confines of the human body tissues and elicits a clinical response. This application has advanced enough so that other forms of intervention may be initiated, such as therapy. Once again, MRI is this type of technology, consisting of reading, interpreting and visualization of the individual hydrogen atomic frequency. Radiofrequency (RF) has also been used to excite proton spins in the body and to receive MRI signal from the body.

### EMF at the cellular level

**Figure 1:**
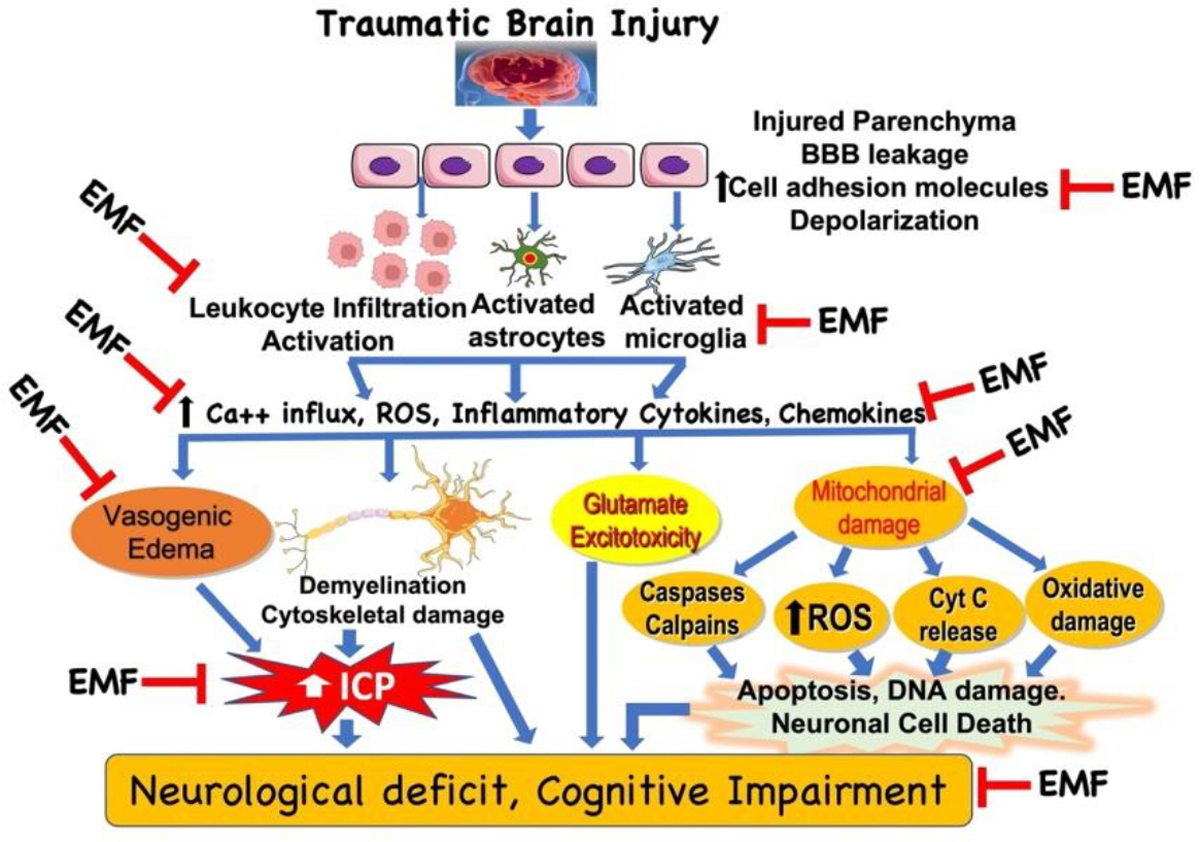
Potential targets in the clinical effect of electromagnetic field (EMF) in traumatic brain injury. Based on the limited literature under *in vitro* and *in vivo* conditions, EMF can attenuate various molecules, cellular and subcellular pathways involved in the pathophysiology of traumatic brain injury. However, detailed analyses of the biophysical mechanisms underlying the effects of EMF are unclear and warrant further investigation. Additionally, it is critical to examine the beneficial effect of EMF in clinically relevant experimental model to extrapolate the findings to clinical situation. BBB, blood brain barrier; ICP, intracranial pressure; ROS, reactive oxygen species.

It is beyond the scope of this article to highlight all clinical effects of EMF and discuss the wide range of proven cellular and subcellular responses to different Extremely Low Frequency (ELF) magnetic fields. These have been reviewed elsewhere and in the Proceedings of the 1st World Congress on the topic [9]. Effects range from changes in reactive oxygen species and intracellular calcium, to modified receptor and messenger behavior, to increased synthesis and degradation (Figure 1).

Highly specific alterations in transcription and translation have been reported, in which the energetic patterns of different fields (e.g., pulse shape and sequencing, frequency characteristics, amplitude, and spatial orientation, among other factors) produce junctional “signatures”. These and other data strongly re-enforce the frequency “windows” and thresholds for bioeffects in which classic dose responses may not exist. Furthermore, data are emerging which indicate a direct interaction between the EMF and a gene without a cascade of biochemical mediated signaling messenger events being initiated at the plasma membrane or in the cytoplasm [10]. In such instances, isolated chromosomes, devoid of cell or nuclear envelopes, respond to field exposure. The mechanisms behind this behavior are moot but may involve resonance effects on ion counter charge at specific loci on the DNA molecule itself. The pattern of bio responses to field exposure depends not only on cell type, its state of function, and its tissue envelope, but also on specific energetic characteristics of the magnetic field. Given this complex situation, it is appropriate to address steps, which led to specifications. The most widely studied condition is bone remodeling. When bone and many other structural tissues are mechanically deformed, they become electrically charged because of piezoelectric, electric, and electrokinetic properties [11]. The amplitude and frequency content of the resultant voltage waveforms reflect both the velocity and magnitude of the deflection. For physiologic loading, voltages between 10 mV and 1 mV/cm are produced with a frequency content predominantly in the range of <1 Hz to 100 Hz or greater, inductively coupled, appropriately congruent, time-varying magnetic fields. The cell does not seem to make a distinction between the sources of the field, only its “informational” content. In fact, ELF magnetic fields can prevent the bone loss that normally occurs during immobilization, bed rest, or weightlessness. These states diminish mechanical deformation, thereby reducing endogenous fields in the microenvironment of the cell. Armed with the voltage patterns, nature appears to communicate instructions to bone, dynamic magnetic fields were designed to produce similar waveforms via inductive coupling [9].

While the biophysical mechanisms underlying these effects are not well understood, several hypotheses have been suggested (Figure 1). The most widely discussed hypothesis (extrapolated from the in vitro data) relates to the intracellular electrophoretic redistribution of molecules. An electric field may alter the membrane potential asymmetrically, thereby perturbing growth-controlling transport processes across the membrane. Thus, the intra-axonal and perhaps, more generally, the intracellular migration of growth-related molecules and certain receptors to key locations within the cell may be a possible explanation for the observed phenomena [12]. Todorov, Yogev, and Qi, documented the effect of PEMF in neurons. They have documented the persistence of connection for 24 hours after axon reconnection and examined the physiology of the reconnected axons. In single cell systems, cell membrane apposition and the magnitude of the field delivered to the membranes are critical parameters affecting cell fusion yields. The functional recovery of neurological systems, such as severed spinal cord and peripheral nerve, depends on the integrity of the axons transmitting information across the injured segment. Axon reconnection offers the promise of rapid return of axon integrity, a necessary condition for the functional recovery of these systems [13]. Direct current (DC)-induced electric fields ranging from low levels of nanovolt per centimeter to high levels of volt per centimeter evoke neurite out-growth that is significantly greater than that observed in control cultures. Moreover, this growth is consistently oriented to the cathode. Electric pulses picoamp per microvolt have been applied focally near growth cones of Xenopus neurons and noted directional growth toward the negative (sink) electrode. Both steady and pulsatile fields were found to be effective in promoting directed neurite outgrowth despite the endogenous occurrence of pulsatile fields. Studies on pulsed electromagnetic fields (PEMF) are fewer in number but are also effective in stimulating growth and upregulating neurotransmitter release. Since it appears that the neurons respond vigorously to the electric fields specifically, it is likely that the currents induced by PEMF, rather than the magnetic fields themselves, are responsible for growth and functional changes [14]. However, as discussed below, and contrary to this citation, the magnetic field is involved as well. A combined AC (850 microGuass)-DC (1200 microGauss (0.12 T)) electromagnetic field has been used to examine cellular alternations of C-6 glioma cell line with 48 and 96-hr exposure. After 48-hr exposure, the cell number decreased 75% compared to the control group. Interestingly, the cell number recovered to 50–60% of the control after 96-hr exposure. There was a 3-fold increase in the average cellular volume after EMF exposure. Analyses using the electron microscope revealed the expansion of the cell diameter in association with depletion of mitochondria, outnumbered lysosomes and multivesicular bodies, and disrupted Golgi apparatus in the experimental group. Bohr demonstrated that the accelerated shift of calcium ions into cells may potentially evoke cytosolic protease, lipase, or catalase, with secondary cytoplasmic organelle injury. Further exploration into the calcium related events on AC-DC field exposure might provide an impetus in unraveling possible neuroprotective strategies in this aspect [15].

### Effects of EMF, PEMF and RF on Vital Functions in Experimental Animals and Human

Many previous studies in peripheral nerve injury models indicate that locally applied weak electric fields can promote axonal regeneration. Using direct current, this galvanotropism is likely to be specific for the cathode source since growth does not advance towards the anode. This principle has been applied in experimental injury of the mammalian CNS, in which there is relatively little or no axonal elongation after injury. The local application of electric fields appears to promote axonal process outgrowth towards the cathode in damaged guinea pig spinal cord, and to promote recovery of function in damaged rat spinal cord. In damaged rat optic nerve, which supports little or no post-traumatic regeneration, cathodes oriented distal to crushed axons can promote substantial axonal process outgrowth for distances of about 3 mm. These observations prompted the investigation of galvanotropism after partial denervation of the hippocampus. The results are consistent with the hypothesis that cathode current can modulate long term effects of injury to this structure. A galvanotropic device (American BioInterface Corporation, New York, NY) has been utilized to deliver a chronic source of weak direct current. The wires led from the opposite ends of the pad and were connected to the cathode or anode terminals of a power supply. The wires were designed so that they spiraled under the pad, each providing a surface area of approximately 0.5 mm^2^. The power supply consisted of a 1.4 V silver oxide hearing aid battery (#36; Eveready, Danbury, CT) and a 1 M ohms resistor in series with the anode, which delivered 1.5 mA to the tissue. The power supply was encased in several layers of medical grade epoxy, which was covered by a SilasticTM layer [16]. As alluded to above, the PEMF responses may not be limited to electrical stimulation and in fact responses may be to an RF field. In a more refined system, the exact frequencies and/or magnetic flux may be determined which can then be used to treat the damage. The technology exists to measure the natural EMF and frequency in the healthy and diseased state. This EMF or a derivation such as RF, corrected for the healing process, is then generated to get the desired outcome. PEMF provides complex information not restricted to amplitude, pulse width and frequency. The ability to deliver multiple pulses has important neurophysiological ramifications. The first demonstration that repetitive TMS (rTMS) could produce dramatic effects on brain function was used to produce speech arrest with stimulation over the motor speech (Broca’s) area, lasting only for the duration of stimulation. No such effects on neural processing had ever been produced with single TMS pulses. Pascual-Leone elucidated some of the neurophysiology of rTMS and RF. They showed that low stimulation frequencies evoked consistent motor evoked potentials (MEPs) in the muscles targeted by the area of stimulated cortex. As the frequency of the stimulation was increased, the stimuli began to influence each other. At frequencies of about 10 Hz, the stimuli reinforced each other. At 20 Hz, every other stimulus came at a time when the corticospinal system was still inhibited by the preceding pulse. This period of inhibition after a TMS pulse usually lasts about 100 ms. As a result, 20 Hz stimulation caused a high degree of synchrony of firing in the corticospinal tract. Because every second pulse could recruit no neurons, the third pulse found many neurons free of inhibition. The net motor output produced an alternating pattern of very small and extremely large motor evoked potentials (MEP). In this way, RF patterns of neuronal activity imposed by rTMS disrupt the normal function of the areas being stimulated. Sustained alterations in neural function and levels of neuronal activity likely underlie the capacity of rTMS to produce reversible functional lesions such as speech arrest [17]. RTMS and RF can produce seizures when stimuli are administered at high rates and intensities or when stimulus trains are administered close together. TMS has been used to precipitate seizures in epileptic patients undergoing presurgical stimulation to map language areas and has produced seizures in normal volunteers [18]. None of the individuals developed any neurological sequelae. During repetitive stimulation of the hand area of motor cortex, it became apparent that at relatively high frequencies of stimulation (> 5 Hz), activity would spread from a single hand muscle to other hand muscles, and from there to more proximal muscles when the intensity of the stimulation was high enough. This represented intracortical spread of excitation and might be an early precursor of epileptic seizure or in the opposite as the spreading depolarization of neuronal injury. Spread of excitation in the primary motor cortex thus provides an indicator of the potential epileptogenicity of rTMS and RF in individual subjects. The primary motor cortex appears to be the most epileptogenic area of the cortex except for the mesial temporal area, which, by virtue of its depth, is inaccessible to rTMS with currently available devices, but not inaccessible to RF. Because the susceptibility to cortical excitation with TMS varies widely among individuals for unknown reasons, the intensity of stimulation must always be chosen with respect to the threshold of an individual subject to produce a motor evoked response. Single-pulse TMS does not appear to affect long-term memory. At the same time, it is proving to be a useful probe of short-term or working memory. Pascual-Leone and co-investigators used rTMS to map the cortical representations of muscles involved in performing a serial reaction time test. As subjects became more familiar with the test and developed implicit knowledge of the repeating pattern, there was a progressive enlargement of the cortical representation of the motor areas involved in the task. After subjects explicitly knew there was a repeating pattern, the cortical representation shrank, demonstrating rapid functional plasticity of cortical regions involved in learning and transfer of knowledge from implicit to explicit. This same group has shown that rTMS over the left mid temporal or bilateral dorsofrontal cortex, at 0 and 250 ms latencies, resulted in impaired recall of a list of recently learned words, demonstrating an effect on working memory [19].

PEMF has been therapeutic in several clinical conditions: (i) mirror movements, a congenital disorder of motor control; (ii) amputations, a typical massive restrictive deafferentation in the peripheral nervous system, (iii) traumatic spinal cord injury leading to paraplegia, and (iv) hemispherectomy for a massive brain lesion. The general hypothesis motivating these experiments was that neural damage in the form of congenital disorders of motor control and lesions in the peripheral and central nervous systems could induce changes in the relationship between cortical motor representation areas and their target muscles. Magnetic stimulation, being less painful than electrical stimulation, is now a useful tool in unveiling these phenomena in humans [17]. Transcranial motor cortex stimulation can serve as an assessment tool for studying the effects of various interventions employed to enhance recovery after spinal cord injury, such as the application of 4-aminopyridine (4-AP), a potassium channel blocker. By blocking exposed potassium channels on spinal cord long tract axons that have been rendered nonfunctional due to demyelination, conduction through the injury zone may be improved. In a group of six spinal cord injury subjects with temperature-dependent central conduction deficits, intravenous application of 4-AP brought a reduction of latency and an increase in amplitude of MEPs in four subjects. Furthermore, two of these subjects also showed improvement in volitional motor unit recruitment in muscles below the level of the lesion. These findings provided conclusive evidence of improved conduction because of a pharmacological intervention to selectively block voltage-gated potassium channels, and thus prolonging action potentials to increase the release of neurotransmitters at the neuromuscular junction and improving conduction in the spinal cord [8]. Since the pathophysiology of neuronal injury may differ between the spinal cord, peripheral nerve, neocortex, and striatum, different timings, and duration of PEMF and RF application are required before concluding that such treatment may be beneficial for cerebral ischemia in brain injury. Young, in 1984, found that a high-frequency pulsed field applied to contused feline spinal cords within 4 hours of injury decreased the accumulation of calcium in the core area of injury and enhanced somatosensory evoked potentials (SSEP) and recovery of motor and sensory function [20]. Zienowicz 1991, demonstrated enhancement of functional recovery in a rat model of peripheral nerve transection combining PEMF exposure with delayed surgical repair [21]. Furthermore, Sisken in 1989, was able to stimulate sciatic nerve regeneration in rats after a crush lesion using a low-frequency PEMF or RF [14]. Rappaport and Young reported on the effect of PEMF on calcium tissue changes in a rat model of focal cerebral ischemia. Rats were treated with a high-frequency PEMF for 2 hours following the onset of focal ischemia, and no differences in brain tissue calcium content were discerned supporting the beneficial effect of PEMF [22]. The effect of sinusoidal magnetic field stimulation on regeneration of the rat sciatic nerve was studied. Rats were exposed, after crush lesioning of the nerve, between a pair of Helmholtz coils to a 50Hz magnetic field of 0.2 millitesla (mT) or 0.4 mT, respectively. Regeneration of the sciatic nerve was measured by the “pinch test,” or by immunocytochemical staining for neurofilaments 1 to 6 days after the crush lesion. Intermittent stimulation (4 hours per day) at 0.2 mT did not affect regeneration, while continuous stimulation with the same field enhanced regeneration distances measured at days 1, 2, and 3. Intermittent stimulation with 0.4 mT increased regeneration distances in 3-day regenerated nerves. In the rats exposed continuously to 0.4 mT regeneration was higher in all groups (1, 2, 3, 4, and 6 days). This field enhanced the regeneration velocity by 21%. Pretreatment for 7 days, with continuous stimulation either at 0.2 mT or at 0.4 mT, did not affect regeneration of the sciatic nerve after a crush lesion [23]. These findings support the role of continuous sinusoidal magnetic field stimulation following crush injury in nerve regeneration. However, careful studies are warranted to support these findings in clinically relevant large animal models. The literature is overflowing with studies demonstrating the effects of PEMF. Brain plasticity occurs 2–4 months after stroke in 18 patients treated with TMS [24]. PEMF increased bone mRNA of BMP-2 and BMP-4. Its effect was related to the duration of the PEMF [25]. PEMF have been used in a single cell system, electric fields of 1–5 kV/cm at nanosecond (ns) to microsecond duration, to coerce macromolecules into a cell. Earthworm axons reconnect at 80–200 Volts, 10–100 microsecond duration. In this system the anode was rostral and the cathode caudal at 200–300 micrometers, with the electric field perpendicular to the cell [13]. All the above studies support the beneficial effect of EMF, PEMF and RF on nerve regeneration, antioxidant activity and many vital functions in experimental animals and human. In the following section, we will discuss the effect of EMF at the cellular level.

EMF has been demonstrated to have potential beneficial effects in neurological recovery. There has been a plethora of both in vivo and in vitro studies that have demonstrated the effects of EMF treatment at different points of the ischemia (Figure 1).

### In vitro Effects of EMF

Nanometer energy can delay neuronal degeneration, rescue marginal cells, and facilitate axonal regeneration. Of course, laser energy can be lethal in high doses. Laser energy at 337.1 nm, one millijoule and 10 Hz, stimulates neurotransmitter release, release of superoxide dismutase and has an anti-inflammatory effect. Yew showed an increase of RNA and protein synthesis, while Fork stimulated the sodium/potassium pump at 488 nm, and Mester stimulated a 162% increase in acetylcholine at 694nm [26–28]. Exposure of cells to relatively low intensity, pulsed, low frequency electromagnetic fields can cause transient augmentation of mRNA [29]. There have been effects demonstrated on key mechanisms of apoptosis, the generation of nitrogen and oxygen reactive species, inflammation, and neuronal protection [30, 31]. Grant et al reported as reduction in edema by 65% in a rabbit model of ischemic stroke, with less neuronal damage in neocortical and striatum regions [32]. Additionally, EMF is a potent inducer of angiogenesis and modulation of microcirculation [33]. EMF has been shown to target processes underlying ischemia after stroke or TBI. To date, studies have been done on diverse cell types, ranging from neurons and microglia to endothelial cells and astrocytes. Astrocytes are the predominant cell type in the brain, have a vital role in the homeostasis, and along with microglia, mediate inflammatory signals. Vincenzi et al 2020 demonstrated PEMF induced protective results in astrocytes, which links astrocytes, stroke, and EMF [34]. Furthermore, PEMF exposure increased vascular endothelial growth factor (VEGF) released in type 1321N1 astrocytes. There have been effects noted in the regulation of apoptosis as well. Gessi et al utilized PEMF and demonstrated decreased antiapoptotic B-cell lymphoma 2 (Bcl-2) molecule and proapoptotic Bcl-2-associated agonist of cell death (BAD). These results were like those reported by other investigators. PEMF stimulates the p38 kinase cascade, with 70 kilodalton heat shock protein (HSP70), cAMP response element-binding protein (CREB), Brain-Derived Neurotrophic Factor (BDNF) being recruited, which ultimately leads to increased levels of antiapoptotic Bcl-2 and simultaneous decrease of apoptotic BAD [35]. Additionally, PEMF activates the BDNF/Tropomyosin receptor kinase B (TrkB)/Protein kinase B (Akt) pathway, which leads to phosphorylation of BAD, which in turn binds to B-cell lymphoma-extra-large (Bcl-xL) and thus decreased Bcl-2-associated X protein (Bax), Bad, and Cas3. These authors noted decreased levels of interleukin-1 (IL-1b) and matrix metallopeptidase 9 (MMP 9) in the periinfarct area of mice suffering from ischemic stroke. PEMF has effects on the endocannabinoid system, employing extracellular signal-regulated kinases (ERK) signaling. PEMF reduced glutamate induced excitotoxicity and with increased viability of cells, and modulation of the endocannabinoid system [34]. These effects were correlated with increased levels of N-arachidonylethanolamide and 2-arachidonoylglycerol.

### In vivo effects of EMF

Motor cortex electrical stimulation in humans is effective for the treatment of central post-stroke pain and trigeminal neuropathic pain. EMF has been shown to promote the activation of anti-inflammatory cascade, while also decreasing pro-inflammatory cytokines. There have been several studies that have shown different genes pertaining to inflammation and apoptosis that are affected by PEMF. Pena-Phillipes et al demonstrated in mice with distal MCA occlusion (dMCAO), PEMF downregulated 19 proinflammatory genes, including IL-1, tumor necrosis factor (TNF) super family genes. Concomitantly, they found an upregulation of interleukin-10 (IL-10) and interleukin-11 (IL-11). This remarkably coincided with a reduction infarction volume and edema. PEMF also attenuated inflammation and hypoxia induced injury across cell lines, resulting in reduced proinflammatory cytokines TNF-α, IL-1β, interleukin-6 (IL-6) and interleukin-8 (IL-8) [34]. Similarly, Merighi and colleagues showed protective effects of PEMF on microglia cells exposed to LPS-induced inflammatory conditions, and described a reduction in TNF-α, IL-1β, and IL-6 [36]. Interestingly, these authors suggested that the protective effect of PEMF was mediated by the recruitment of c-Jun N-terminal Kinase 1/2 (JNK1/2) mitogen-activated protein kinases (MAPK), as the inhibition of JNK1 reversed the protective effects from PEMF on TNF-α and IL-1β production. PEMF in this case reduced the cellular functions of microglial as it pertains to reactive oxygen species (ROS) production, cellular invasion, and phagocytosis. SEMF has been shown to decreased levels of superoxide in addition to lowering levels of intracellular reactive oxygen species (ROS), nitric oxide (NO), lipoprotein (LP), and calcium, while also increasing superoxide dismutase (SOD) and NO [37]. Duong Kim showed that under hypoxia, SEMF decreased intracellular calcium, and ROS production. These findings were echoed by Pessina et al, who showed a reduction of intracellular calcium in astrocytoma after PEMF [38]. Most studies have shown that EMF does in fact induce NO [39, 40]. NO is produced by endothelial cells after SEMF is applied to cells. When NOS was inhibited, the positive effect of ELF-MF in rats with stroke was inhibited [41]. It is important to note that NO is synthesized in endothelial, neuronal, and inducible subtypes. While NO from endothelium results in vasodilation with increase in blood flow to ischemic brain regions, neuronal nitric oxide synthase (nNOS) and inducible nitric oxide synthase (iNOS) on the other hand, may have deleterious effects. Therefore, it will be prudent to conduct further research to elucidate how the various isoforms of NOS are activated by EMF. Based upon the above data, PEMF stimulates RNA and protein synthesis, brings molecules into cells, rescues cells from anoxia, leads to axon regeneration and improves functional recovery after stroke. The effects of EMF on the oxidant-antioxidant defense system have been debatable. This could be due to the exposure of variable strengths and wavelength whereby high dose of EMF exposure triggers oxidative stress in several tissues, but at lower doses it may reduce oxidative stress by inhibiting the generation of reactive oxygen species and thus preventing the damage of cellular components, including proteins, lipids, and DNA. However, the underlying mechanisms are still unclear and warrant further studies.

### Potential Cellular Targets for EMF Modulation

#### Spreading depolarization

Spreading depolarizations refer to waves of mass depolarization in neuronal gray matter and serve as markers and responses to pathological events. These depolarizations are highly varied and involve multiple molecules, earning them the nickname “brain tsunami”, resulting in the release of large amounts of potassium and neurotransmitters, while also leading to the influx of sodium, calcium, and water into neuronal cells. This process increases metabolic demand, potentially changing blood flow to the brain, and disrupting the blood-brain barrier. Spreading depolarizations may also impact gene expression and alter the ultrastructure of the brain [42]. Since it spreads beyond the injury, it affects healthy tissue too. One hypothesis worth exploring is if EMF applied over injured tissue will limit superoxide dismutase and result in improved neurological outcomes. Some studies suggest that physiological optimization strategies and/or pharmacologic therapy could inhibit superoxide dismutase in malignant hemispheric syndrome (MHS) patients, and thereby limit edema and infarct progression, there would be reason to believe that EMF might be able to exert the same effect [43].

#### Biomarkers to monitor the effect of EMF

Several potential biomarkers of brain tissue damage, including the structural proteins glial fibrillary acidic protein (GFAP) and S100 Calcium Binding Protein B (S100β), and the matrix protein MMP-9, show promise. Combining multiple biomarkers in panels can improve the accuracy of ischemic stroke diagnosis. Although unbiased “omics” approaches can identify a broad range of genes, proteins, and metabolites, their high cost makes them unlikely to be the preferred detection method [44]. Whereas sphenopalatine ganglion stimulation has used the release of NO, acetylcholine and BIP from parasympathetic innervation to cerebral blood circulation, so, too, may these markers be monitored as EMF is applied over similar stroke morphologies [45]. Certain biomarkers are released at certain times, which may imply that a temporal application of EMF would benefit patients with brain injury at different times, or at different intervals. The STAIR XI workshop discussed tissue imaging techniques that can identify stroke patients with both core and salvageable brain tissue. They referred to this approach as a “tissue window” for selecting patients for treatment. The elements of the neurovascular unit have varying vulnerability and evolve over different time scales in different brain regions. STAIR proposed the term “target window” to suggest therapies that target different elements of the neurovascular unit at different times [46]. Other studies imply that inhibition of certain expressed molecules may be beneficial in enhancing recovery after brain injury. Patabendige et al, discussed the role of aquaporin 4 (AQP 4) and its polarized expression on astrocytic end feet are important for bidirectional water flux in the neurovascular unit. Reduction of AQP4 expression and translocation from astrocytic end feet leads to cytotoxic edema, and inhibitors of this translocation could reduce cytotoxic edema and potentially be neuroprotective for ischemic stroke [47]. If EMF shows promise in also acting as an inhibitor of the actions of certain molecules, then the potential to provide similar neuroprotective benefits is also possible. Newer studies are even demonstrating more biomarkers that can be tracked as evidence of brain injury and ischemia, that may serve as indicators that EMF is indeed leading to enhanced healing mechanisms at the cellular level.

### Evaluating Cognitive Outcomes in Humans

There are several studies that have used cognitive outcomes to measure the effects and sequalae of various brain injuries, both ischemic and traumatic. The LACunar Intervention Trial-2 (LACI-2) was designed to determine the feasibility of a larger trial that would test the effectiveness of cilostazol and/or isosorbide mononitrate (ISMN). The purpose of LACI-2 was to confirm outcome event rates required to power a phase 3 trial and test participant recruitment and retention in follow-up, drug tolerability, and safety. The LACI-2 trial used Montreal Cognitive Assessment (MOCA) and Trails V, along with hematologic and biochemical markers [48]. During the International Stroke Conference held in Dallas, Texas from February 8–10, 2023, researchers presented the results of a phase 1a/2b study. This double-blind, randomized controlled trial evaluated the effectiveness of ApTOLL, an experimental drug, in combination with endovascular therapy (EVT) in patients with acute ischemic stroke. The study specifically focused on patients who had confirmed large vessel occlusion and were eligible for reperfusion therapies, such as EVT, with or without intravenous recombinant tissue plasminogen activator [49]. These researchers used changes in National Institutes of Health Stroke Scale score (NIHSS) and modified Rankin Scale (mRS) as primary cognitive endpoints.

## Conclusion

Based on the evidence reviewed in this review paper, exposure to electromagnetic frequencies appears to play a beneficial role in optimizing cellular mechanisms for healing after brain trauma. While further research is needed to fully understand the underlying cellular and molecular mechanisms and develop the most effective personalized protocol to determine the optimal frequency, duration, and intensity of exposure, the current findings suggest that electromagnetic therapy may hold promise as a potential non-invasive treatment for traumatic brain injury. Future research in clinical models and the response to EMF will depend on a variety of accepted and experimental biomarkers currently in use to evaluate response to treatment, as well as cognitive outcomes. It is important to note, however, that more research is needed to fully understand the potential benefits and risks of electromagnetic therapy for brain trauma. As with any treatment, it is crucial to carefully weigh the potential benefits against the potential risks, and to consult with a healthcare professional before beginning any new therapy.

## Data Availability

Not applicable since the information is gathered from published articles.

## References

[1] Nordhausen CT, Maynard EM, Normann RA. Single unit recording capabilities of a 100 microelectrode array. Brain Res 726 (1996): 129–140. 8836553

[2] Rousche PJ, Normann RA. Chronic recording capability of the Utah Intracortical Electrode Array in cat sensory cortex. J Neurosci Methods 82 (1998): 1–15. 10223510 10.1016/s0165-0270(98)00031-4

[3] Benabid AL, Benazzous A, Pollak P. Mechanisms of deep brain stimulation. Mov Disord 17 (2002): S73–74. 11948758 10.1002/mds.10145

[4] Kellaway P, Gol A, Proler M. Electrical activity of the isolated cerebral hemisphere and isolated thalamus. Exp Neurol 14 (1966): 281–304. 4951843 10.1016/0014-4886(66)90115-4

[5] Teshome AK, Kibret B, Lai DTH. A Review of Implant Communication Technology in WBAN: Progress and Challenges. IEEE Rev Biomed Eng 12 (2019): 88–99. 29994664 10.1109/RBME.2018.2848228

[6] Merton PA, Morton HB. Stimulation of the cerebral cortex in the intact human subject. Nature 285 (1980): 227–227. 7374773 10.1038/285227a0

[7] Barker AT, Jalinous R, Freeston IL. Non-invasive magnetic stimulation of human motor cortex. The Lancet 325 (1985): 1106–1107. 10.1016/s0140-6736(85)92413-4 2860322

[8] McKay WB, Stokic DS, Dimitrijevic MR. Assessment of Corticospinal Function in Spinal Cord Injury Using Transcranial Motor Cortex Stimulation: A Review. J Neurotrauma 14 (1997): 539–548. 9300564 10.1089/neu.1997.14.539

[9] Bassett CAL. Beneficial effects of electromagnetic fields: Beneficial Effects of Electromagnetic Fields. J Cell Biochem 51 (1993): 387–393. 8496242 10.1002/jcb.2400510402

[10] Goodman R, Bassett CAL, Henderson AS. Pulsing Electromagnetic Fields Induce Cellular Transcription. Science 220 (1983): 1283–1285. 6857248 10.1126/science.6857248

[11] Hastings GW, Mahmud FA. Electrical effects in bone. J Biomed Eng 10 (1988): 515–521. 3070168 10.1016/0141-5425(88)90109-4

[12] Politis MJ, Zanakis MF, Albala BJ. Mammalian Optic Nerve Regeneration Following the Application of Electric Fields. J Trauma Acute Care Surg 28 (1988). 10.1097/00005373-198811000-00005 3184216

[13] Todorov AT, Yogev D, Qi P, Electric-field-induced reconnection of severed axons. Brain Res 582 (1992): 329–334. 1393555 10.1016/0006-8993(92)90151-x

[14] Sisken BF, Walker J, Orgel M. Prospects on clinical applications of electrical stimulation for nerve regeneration: Nerve Regeneration and Electricity. J Cell Biochem 51 (1993): 404–409. 8496243 10.1002/jcb.2400510404

[15] Patchana T K Agrawal D, Connett D, Immunomodulatory Effect of Electromagnetic Field in the Treatment of Traumatic Brain Injury. J Biotechnol Biomed 06 (2023). 10.26502/jbb.2642-91280069 PMC9977325 36865683

[16] Politis MJ, Zanakis MF. Treatment of the damaged rat hippocampus with a locally applied electric field. Exp Brain Res 71 (1988). 10.1007/BF00247539 3416955

[17] George M, Wassermann E, Post R. Transcranial magnetic stimulation: a neuropsychiatric tool for the 21st century. J Neuropsychiatry Clin Neurosci 8 (1996): 373–382. 9116472 10.1176/jnp.8.4.373

[18] Narayana S, Gibbs SK, Fulton SP, Clinical utility of Transcranial Magnetic Stimulation (TMS) in the presurgical evaluation of motor, speech, and language functions in young children with refractory epilepsy or brain tumor: Preliminary evidence. Front Neurol 12 (2021):650830. 34093397 10.3389/fneur.2021.650830 PMC8170483

[19] Pascual-Leone A, Tarazona F, Keenan J, Transcranial magnetic stimulation and neuroplasticity. Neuropsychologia 37 (1998): 207–217. 10.1016/s0028-3932(98)00095-5 10080378

[20] Young W Pulsed electromagnetic fields alter calcium in spinal cord injury. Cent Nerv Sys Trauma 1 (1984): 100.

[21] Zienowicz RJ, Thomas BA, Kurtz WH, A multivariate approach to the treatment of peripheral nerve transection injury: the role of electromagnetic field therapy. Plast Reconstr Surg 87 (1991): 122–129. 1984256 10.1097/00006534-199101000-00019

[22] Rappaport ZH, Young W. Effect of pulsed electromagnetic fields on calcium tissue changes in focal ischaemia. Neurol Res 12 (1990): 95–98. 1974708 10.1080/01616412.1990.11739924

[23] Rusovan A, Kanje M. Stimulation of regeneration of the rat sciatic nerve by 50 Hz sinusoidal magnetic fields. Exp Neurol 112 (1991): 312–316. 2029930 10.1016/0014-4886(91)90132-v

[24] Cicinelli P, Traversa R, Rossini P. Post-stroke reorganization of brain motor output to the hand: a 2–4 month follow-up with focal magnetic transcranial stimulation. Electroencephalogr Clin Neurophysiol Mot Control 105 (1997): 438–450. 10.1016/s0924-980x(97)00052-0 9448645

[25] Bodamyali T, Bhatt B, Hughes F, Pulsed electromagnetic fields simultaneously induce osteogenesis and upregulate transcription of bone morphogenetic proteins 2 and 4 in rat osteoblastsin vitro. Biochem Biophys Res Commun 250 (1998): 458–461. 9753652 10.1006/bbrc.1998.9243

[26] Yew DT, Wong SL, Chan Y wa. Stimulating effect of the low dose laser–A new hypothesis. Cells Tissues Organs 112 (1982): 131–136. 10.1159/000145504 6179384

[27] Fork RL. Laser stimulation of nerve cells in Aplysia. Science 171 (1971): 907–908. 5541653 10.1126/science.171.3974.907

[28] Mester E, Mester AF, Mester A. The biomedical effects of laser application. Lasers Surg Med 5 (1985): 31–39. 3982191 10.1002/lsm.1900050105

[29] Litovitz T, Montrose C, Goodman R, Amplitude windows and transiently augmented transcription from exposure to electromagnetic fields. Bioelectromagn J Bioelectromagn Soc Soc Phys Regul Biol Med Eur Bioelectromagn Assoc 11 (1990): 297–312. 10.1002/bem.2250110406 2285415

[30] Funk RHW, Monsees T, Özkucur N. Electromagnetic effects – From cell biology to medicine. Prog Histochem Cytochem 43 (2009): 177–264. 19167986 10.1016/j.proghi.2008.07.001

[31] Morris CE, Skalak TC. Acute exposure to a moderate strength static magnetic field reduces edema formation in rats. Am J Physiol-Heart Circ Physiol 294 (2008): H50–H57. 17982018 10.1152/ajpheart.00529.2007

[32] Grant G, Cadossi R, Steinberg G. Protection against focal cerebral ischemia following exposure to a pulsed electromagnetic field. Bioelectromagnetics 15 (1994): 205–216. 8074737 10.1002/bem.2250150305

[33] Li RL, Huang JJ, Shi YQ, Pulsed electromagnetic field improves postnatal neovascularization in response to hindlimb ischemia. Am J Transl Res 7 (2015): 430–444. 26045885 PMC4448185

[34] Moya Gómez A, Font LP, Brône B, Electromagnetic field as a treatment for cerebral ischemic stroke. Front Mol Biosci 8 (2021): 742596. 34557522 10.3389/fmolb.2021.742596 PMC8453690

[35] Gessi S, Merighi S, Bencivenni S, Pulsed electromagnetic field and relief of hypoxia-induced neuronal cell death: The signaling pathway. J Cell Physiol 234 (2019): 15089–15097. 30656694 10.1002/jcp.28149

[36] Merighi S, Gessi S, Bencivenni S, Signaling pathways involved in anti-inflammatory effects of Pulsed Electromagnetic Field in microglial cells. Cytokine 125 (2020): 154777. 31400640 10.1016/j.cyto.2019.154777

[37] Naarala J, Kesari KK, McClure I, Direction-dependent effects of combined static and ELF magnetic fields on cell proliferation and superoxide radical production. BioMed Res Int 2017 (2017): 5675086. 28497056 10.1155/2017/5675086 PMC5405400

[38] Pessina G, Aldinucci C, Palmi M, et al. Pulsed electromagnetic fields affect the intracellular calcium concentrations in human astrocytoma cells. Bioelectromagn J Bioelectromagn Soc Soc Phys Regul Biol Med Eur Bioelectromagn Assoc 22 (2001): 503–510. 10.1002/bem.79.abs 11568936

[39] Okano H, Masuda H, Ohkubo C. Decreased plasma levels of nitric oxide metabolites, angiotensin II, and aldosterone in spontaneously hypertensive rats exposed to 5 mT static magnetic field. Bioelectromagnetics 26 (2005): 161–72. 15768432 10.1002/bem.20055

[40] Cichoń N, Czarny P, Bijak M, Benign effect of extremely low-frequency electromagnetic field on brain plasticity assessed by nitric oxide metabolism during poststroke rehabilitation. Oxid Med Cell Longev 2017 (2017): 2181942. 29138675 10.1155/2017/2181942 PMC5613626

[41] Font LP, Cardonne MM, Kemps H, Non-pulsed sinusoidal electromagnetic field rescues animals from severe ischemic stroke via NO activation. Front Neurosci 561 (2019). 10.3389/fnins.2019.00561 PMC6593085 31275094

[42] Ayata C, Lauritzen M. Spreading depression, spreading depolarizations, and the cerebral vasculature. Physiol Rev 95 (2015): 953–993. 26133935 10.1152/physrev.00027.2014 PMC4491545

[43] Chau L, Davis HT, Jones T, Spreading depolarization as a therapeutic target in severe ischemic stroke: Physiological and pharmacological strategies. J Pers Med 12 (2022): 1447. 36143232 10.3390/jpm12091447 PMC9502975

[44] Dagonnier M, Donnan GA, Davis SM, Acute stroke biomarkers: are we there yet? Front Neurol 12 (2021): 619721. 33633673 10.3389/fneur.2021.619721 PMC7902038

[45] Hosseini MB, Saver JL. Mechanisms of action of acute and subacute sphenopalatine ganglion stimulation for ischemic stroke. Int J Stroke 15 (2020): 839–848. 32326842 10.1177/1747493020920739

[46] Lyden P, Buchan A, Boltze J, Top priorities for cerebroprotective studies—a paradigm shift: report from STAIR XI. Stroke 52 (2021): 3063–3071. 34289707 10.1161/STROKEAHA.121.034947 PMC8384700

[47] Patabendige A, Chen R. Astrocytic aquaporin 4 subcellular translocation as a therapeutic target for cytotoxic edema in ischemic stroke. Neural Regen Res 17 (2022): 2666. 35662202 10.4103/1673-5374.339481 PMC9165363

[48] Wardlaw J, Bath PMW, Doubal F, Protocol: The Lacunar Intervention Trial 2 (LACI-2). A trial of two repurposed licensed drugs to prevent progression of cerebral small vessel disease. Eur Stroke J 5 (2020): 297–308. 33072884 10.1177/2396987320920110 PMC7538764

[49] Hernandez M, Cotgreave I, Gallego J. A double-blind, placebo-controlled, randomized, phase 1b/2a clinical study of ApTOLL for the treatment of acute ischemic stroke. International Stroke Conference (2023). 10.3389/fneur.2023.1127585 PMC9999729 36908619

